# Conservative approach for a giant prostatic hyperplasia of 1280 ml: a case report and literature review

**DOI:** 10.1093/jscr/rjad422

**Published:** 2023-08-23

**Authors:** Luigi Quaresima, Pietro Tramanzoli, Daniela Fasanella, Andrea Benedetto Galosi, Willy Giannubilo

**Affiliations:** Division of Urology , Civitanova Marche Hospital, Civitanova Marche, Italy; Department of Clinical and Specialist Sciences, Division of Urology, Polytechnic University of the Marche Region Medical School, Ancona, Italy; Department of Life, Health and Environmental Sciences, University of L'Aquila, L'Aquila, Italy; Department of Clinical and Specialist Sciences, Division of Urology, Polytechnic University of the Marche Region Medical School, Ancona, Italy; Division of Urology , Civitanova Marche Hospital, Civitanova Marche, Italy

## Abstract

Benign Prostatic Hypertrophy (BPH) affects at least one-third of men over 60 years. A giant prostatic hyperplasia (GPH) is a prostate enlargement that exceeds 500 g. We present a case of a 72-year-old man with a GPH volume of 1280 ml, referred to our hospital for a worsening of the lower urinary tract symptoms (LUTS), bilateral loin pain and kidney failure. Although the patient had a negligible post-void residual urine, he had bilateral hydronephrosis. The patient was managed conservatory because of a high anesthesiologic risk but a bilateral percutaneous nephrostomy was placed soon due to kidney function worsening. The presence of serious comorbidities and the resolution of the loin pain and the renal failure, achieved first with the nephrostomy and then with periodic replacement of ureteral stents, along with an improvement of the LUTS obtained with medical therapy, have oriented us towards a conservative management of the patient.

## INTRODUCTION

Benign Prostatic Hypertrophy (BPH) affects at least one-third of men over 60 years [1] and its incidence is age related [2]. The term giant prostatic hyperplasia (GPH) refers to prostates larger than 500 g [3]. We present a case of a 72-year-old man with a GPH who presented kidney injury due to bilateral hydronephrosis and no urinary retention.

## CASE REPORT

**Figure 1:**
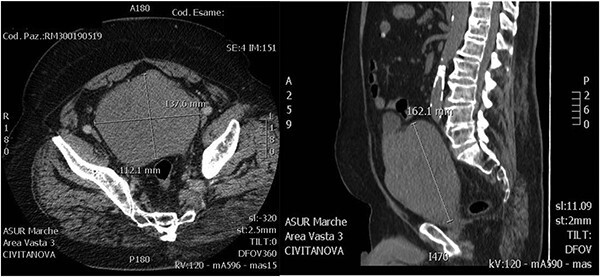
Axial and sagittal CT images.

A 72-year-old man was admitted to the emergency department complaining bilateral flank pain and severe lower urinary tract symptoms (LUTS). His International Prostatic Symptoms Score (IPSS) was 27. His past medical history showed alcohol abuse, stage II chronic kidney disease, atrial fibrillation complicated by pulmonary embolism and stroke. His body mass index was 35. He was on Rivaroxaban therapy. Rectal examination showed a significantly enlarged prostate, with a smooth surface and with no nodularity. He underwent an abdominal ultrasound that showed bilateral grade III hydroureteronephrosis and a huge prostate, with negligible post-void residual urine. Laboratory tests showed a serum creatinine of 2.61 mg/dl, PSA 6.19 ng/ml and no electrolytes imbalance. Uroflowmetry was performed showing a Q max 9 ml/s, Volume void 180 ml and RPM 15 ml. He started therapy with Dutasteride 0,5 mg and Silodosin 8 mg. An abdominal contrast-enhanced computed tomography (CT) scan was performed to evaluate bilateral hydronephrosis, and showed persistent dilatation of the collecting system and ureters bilaterally, with thinning of the bilateral cortical and an estimated prostate volume of 1280 ml (Fig. 1). A percutaneous nephrostomy was placed bilaterally and anterograde pyelography was performed, showing extreme tortuosity of the ureters. Serum creatinine decreased to 1.25 mg/dl 1-month later, and the nephrostomies were closed. A multiparametric magnetic resonance imaging (MRI) of the prostate was performed showing that the peripheral zone was not recognizable and the transition zone was occupied by a huge adenoma. After 3 months of combination therapy, the patient reported improvement in LUTS (IPSS 15, QoL2). Then, he underwent anterograde pyelography that showed poor contrast medium progression. Subsequently, percutaneous nephrostomies were replaced with double J stents. Two months later, a decrease of bilateral hydronephrosis was demonstrated and serum creatinine remained stable at 1.20 mg/dl. After 6 months, patient’s creatinine was 1.22 mg/dl and he was scheduled for retrograde stent replacement with flexible cystoscope. Uroflowmetry was repeated showing a Q max 23.8 ml/s, Volume void 213 ml and RPM 40 ml.

At cystoscopy, the bladder was only partially explorable because of the voluminous intravesical prostatic protrusion. The exam revealed diffuse trabeculation inside the urinary bladder compatible with bladder muscle hypertrophy. The ureteral orifices were strongly lateralized with double J stent protruding from them.

The resolution of the kidney failure and the loin pain, along with an improvement of the LUTS, has corroborated the indication to a conservative management of the patient.

## DISCUSSION

BPH is a frequent cause of bothersome LUTS but in some cases BPH can present with complications such as urinary retention, hydronephrosis or haematuria [5, 6] Giant prostatic enlargement refers to a GPH. GPH was coined by Fishman and Merrill to describe prostate weighing >500 g [7]. So far, there have been described only 15 cases of prostate volume larger than 700 g (Table 1) [2]. The genesis of GPH is not known. However, an over-expression of growth factors combined with the absence or reduction of inhibitory factors have been proposed as possible mechanisms. The mutation of protooncogenes such as *Ras* and *c-erbB-2* may also be involved, developing a continuous cellular proliferation signal or the loss of influence of the p53 suppressor gene through its mutation or deletion, which would allow for abnormal cell proliferation [5]. Two mechanisms have been proposed for hydronephrosis in patients with BPH: first, anatomic obstruction of the ureterovesical junction due to bladder muscle hypertrophy as a result of benign prostatic obstruction; and second, functional compression of the ureterovesical junction, leading to an increase in ureteral resistance through the ureteral tunnel due to bladder over-distension [9]. Surgical intervention is indicated in the presence of LUTS not responding to medical therapy, acute or chronic urinary retention, recurrent gross haematuria, urinary tract infections, renal failure due to hydronephrosis or bladder stones. According to the European Association of Urology (EAU) Guidelines, transurethral surgical resection techniques remain the standard surgical procedure for men with prostate sizes of 30–80 g and bothersome moderate-to-severe LUTS secondary of BPH. However open surgery is recommended for bigger prostates (>80–100 g): among the various techniques available, most surgeons still opt for open prostatectomy. Our case represents the third largest prostate reported in the literature. The largest prostate surgically treated was a 2410 g prostate in a 57-year-old man with LUTS, reported by Pérez *et al*. [8] in 1997. However, Domínguez *et al*. [10] reported a case of a 72-year-old man with MRI diagnosis of a GPH with a prostatic volume of 3987 ml in 2016. This patient required no treatment because he had mild LUTS which remained stable over a period of 10-year follow-up. The treatment options for our patient were limited by his comorbidities: obesity, atrial fibrillation, history of pulmonary embolism and ischemic stroke which featured a high anesthesiologic risk. Open prostatectomy was offered but the patient declined due to the high surgical and anesthesiologic risks. The resolution of symptoms and hydronephrosis with medical therapy and the placement of bilateral ureteral stents have oriented us towards a conservative management of the patient.

**Table 1:** GPH in medical literature

Author	Weight (g) / Volume(ml)
Domínguez *et al.* [9]	3987^*^
Medina-peres *et al.* [8]	2410
Quaresima *et al.* (Current case)	1280^*^
Dincer *et al.* [4]	1090
Aghamir *et al.* [2]	1070
Tolley da *et al.* [2]	1058
Ockerblad [2]	820
Appiah *et al.* [2]	800
Wang *et al.* [2]	800^*^
Maliakal *et al.* [2]	740
Ucer *et al.* [2]	734
Nelson [2]	720
Wroclawski [2]	720
Gilbert [2]	713
Lacy *et al.* [2]	708
Wadstein [2]	705
Lantzius-beninga [2]	705
Khan *et al.* [2]	700
Egote *et al.* [2]	700

^*^Measured in volume (ml) by CT or MRI because prostates were not removed for weighting unlike others

GPH is a rare pathology of the prostate gland. In this study, we report a successful conservative management of a GPH (1280 ml). This may become a viable alternative in patients with GPH in the absence of urinary retention.
